# Distance from human dwellings differentially affects the efficacy of a synthetic cattle urine odour lure to trap malaria vectors

**DOI:** 10.1186/s12936-022-04437-7

**Published:** 2023-01-06

**Authors:** Godfrey C. Katusi, Samwely M. Makayula, Nicodem J. Govella, Ladslaus L. Mnyone, Sharon R. Hill, Rickard Ignell

**Affiliations:** 1 Department of Environmental Health and Ecological Sciences, Ifakara Health Institute, Off Mlabani Passage, 53, Ifakara, Morogoro Tanzania; 2 Department of Microbiology, Parasitology and Biotechnology, College of Veterinary Medicine and Biomedical Sciences, Sokoine University of Agriculture, 3019, Morogoro, Tanzania; 3 Disease Vector Group, Unit of Chemical Ecology, Department of Plant Protection Biology, Swedish University of Agricultural Sciences, 190, 234 22 Lomma, Sweden; 4 Institute of Pest Management, Sokoine University of Agriculture, 3110, Morogoro, Tanzania; 5 School of Life Sciences and Bioengineering, Nelson Mandela African Institution of Science and Technology, Arusha, Tanzania

**Keywords:** Semiochemicals, Mosquitoes, Control, Surveillance, Outdoor

## Abstract

**Background:**

Cost–effective outdoor–based devices for surveillance and control of outdoor mosquito vector populations can substantially improve their efficacy when baited with synthetic human and animal odours. This study aimed at assessing the dose–dependent efficacy of a previously developed synthetic cattle urine odour to lure malaria vectors, and other mosquito species, to traps placed at different distances from human dwellings outdoors.

**Methods:**

The efficacy of the cattle urine odour lure was assessed through a 5 × 5 Latin square design, using two sets of 5 Suna traps placed at either 1.5 m or 5 m from an adjacent human dwelling, in the rural village of Sagamaganga, Tanzania. Each trap was deployed with one of four doses of the synthetic cattle urine odour blend or a solvent control (heptane). Traps were rotated daily so that each dose and control visited each position twice over a period of 20 experimental nights. The relative attractiveness of each treatment dose and control was compared using a generalized linear mixed model for each species caught.

**Results:**

A total of 1568 mosquitoes were caught, of which 783 were anophelines and 785 were culicines. Of the anophelines, 41.6 and 58.3% were primary and secondary vector species, respectively. Unfed and fed females of the primary vector, *Anopheles arabiensis*, were caught dose–dependently, close to human dwellings (1.5 m), whereas unfed, fed and gravid secondary vector *Anopheles pharoensis* females were caught dose–dependently, but at a farther distance from the dwellings (5 m). Females of *Culex* spp. were caught dose–dependently in similar numbers irrespective of the distance from human dwellings.

**Conclusions:**

This study further clarifies the factors to be considered for the implementation of outdoor trapping using the synthetic cattle urine lure to target exophilic and exophagic malaria vectors, for which efficient surveillance and control tools are currently lacking. The findings resulting from this study make significant progress in providing the needed information to overcome the regulatory obstacles to make this tool available for integrated vector management programs, including registration, as well as evaluation and regulation by the World Health Organization.

## Background

Outdoor–based devices are required to complement current surveillance and frontline control tools [1–3], as a result of changes in biting times, and in the composition of outdoor–biting malaria vector species. These factors increasingly contribute to persistent malaria transmission in sub–Saharan Africa and beyond [2, 4–6]. Cost–effective outdoor–based devices for surveillance and control of outdoor mosquito vector populations can substantially improve their efficacy, when baited with synthetic human and animal odours [7–10]. While several synthetic blends mimicking human odour (*e.g.*, Mbita, BG, Ifakara lures) have been developed, these are limited in their deployment, as these only lure host-seeking females, and require the addition of carbon dioxide, which is difficult to procure and prohibitively expensive in remote areas [11–13]. Thus, alternative odour-based lures targeting males and females at other physiological states need to be assessed for their efficacy.

Cattle urine attracts many haematophagous insects [14, 15], with fresh and aged cattle urine demonstrated to attract various physiological stages and species of mosquitoes [16–18]. Adult female malaria vectors use the urine as a supplementary nitrogen–rich meal, enhancing flight mobility, survival and reproductive traits [19]. The drive for locating nitrogen-rich resources by malaria vectors can be harnessed for vector and malaria control, as shown by [20], in which a synthetic cattle urine odour blend was used to lure host seeking, blood fed and gravid females into traps in a rural, malaria endemic region in southern Ethiopia. Further proof concerning the attractiveness of the synthetic cattle urine odour to malaria vectors in different settings with various trap placements relative to human dwellings is, however, needed to confirm and improve its utility as a surveillance and control tool in integrated vector management.

In this study, the efficacy of the synthetic cattle urine odour lure in attracting malaria vectors outdoors was evaluated in the Kilombero Valley, Tanzania, by placing traps close to and away from human dwellings. The perspective of using the synthetic cattle urine odour blend in improving surveillance and control of outdoor biting malaria vectors is discussed.

## Methods

### Study area

**Fig. 1:**
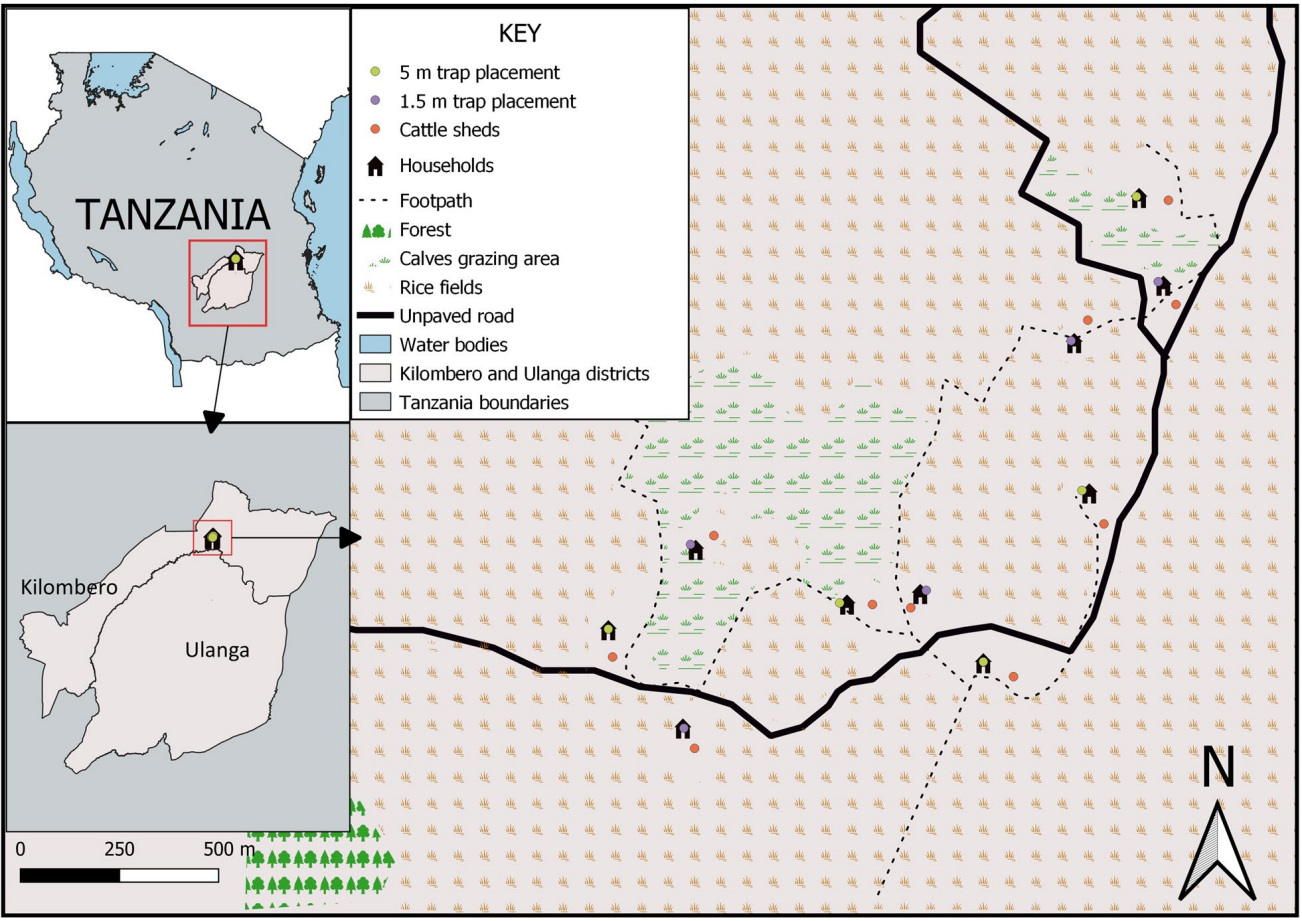
Map showing the location of the Suna traps® next to the human dwellings, in relation to the cattle sheds, in the study village, Sagamaganga, in south-eastern Tanzania

The study was conducted in Sagamaganga village (S 8° 3′ 50.352″ E 36° 47′ 46.254″), Kilombero Valley, south–eastern Tanzania at an altitude of 300 m above sea level (Fig. 1). The average annual temperature ranges between 20 °C and 32 °C, with an annual rainfall between 1200 mm and 1800 mm [21, 22]. The area experiences two main seasons, the wet and dry, which extend from February to June and July to January, respectively. The main economic activities are agricultural, including rice cultivation and livestock keeping. The common domesticated animals are predominantly cattle, goats, sheep, chickens and dogs, with cattle as the most abundant. *Anopheles arabiensis* and *Anopheles coustani* have been identified as the most abundant primary and secondary vectors of malaria, respectively [23]. The malaria prevalence rate in Kilombero valley has decreased from 14% in 2007–2011 [22, 24] to 0.4% in 2019 (Kyeba et al*.*, pers. commun.).

### Selection and characteristics of study households

Ten study households were selected randomly from a register of all houses by using global positioning system coordinates of 152 households in the study village in simple random sampling (Fig. 1) [25]. All study households had mud–brick walls and open eaves. Three households had thatched roofs, while the remaining seven were made of corrugated iron. All selected study households had at least one cattle shed located approximate 50 m to 100 m from the house (Fig. 1). The number of occupants per household varied from one to five. Each household was provided with one-to-three new long-lasting insecticidal nets (Olyset, A to Z Textiles Mills, Arusha, Tanzania) depending on the number of beds. In all the study households, no animals were kept inside the human dwellings, and cooking was exclusively conducted outside.

### Assessing the effect of dose and trapping distance of the synthetic cattle urine odour

The attractiveness of the synthetic cattle urine odour lure [19] against wild mosquitoes was assessed through a repeated 5 × 5 Latin square design, using Suna traps® (Biogents AG, Germany). Two sets of five traps, placed at either 1.5 m or 5 m from the household, were evaluated during each experimental night. The traps were suspended approximately 20 cm above the ground, 50–100 m away from any adjacent cattle shed, and run overnight from 18h00 to 06h00. Each trap was deployed with the synthetic cattle urine odour blend: 7:9:156:156:1:4; 2-cyclohexen-1-one (96% GC grade VWR, Stockholm, Sweden): phenol: *p*–cresol: *m*–cresol: decanal: linalool (> 95.0% GC grade, Sigma Aldrich Sweden AB, Stockholm) [19] at one of four doses (0.1, 0.03, 0.01, 0.003 volume/ volume) or a solvent control (heptane, 97.0% solvent GC grade, Sigma Aldrich). The odour blend and heptane (2 ml) were dispensed via wick dispensers, which allow the release of all compounds in constant ratios throughout the night [26]. Initially, the treatments and controls were randomly assigned to the ten houses, then subsequently rotated nightly in serial order, so that each dose and control visited each trapping position twice over a period of 20 experimental nights. This trial was conducted in four rounds: 5 nights in April, 5 nights in May, and 10 nights in June 2021.

### Adult mosquito identification

Caught anopheline mosquitoes were preserved in 1.5 ml Eppendorf tubes containing silica gel while awaiting further laboratory analyses. The mosquitoes were identified using morphological identification keys [27, 28], and sorted based on sex and physiological state (abdominal status: unfed, fed, semi-gravid and gravid). Molecular species identification, using multiplexed polymerase chain reaction, was performed on all collected *An. gambiae *sensu lato (*s.l*.) (n = 232) [28, 30] and *Anopheles funestus s.l.* (n = 23) [29, 31, 32].

### Statistical analysis

Daily variation in mosquito captures across different doses of the synthetic odour blend were analysed using generalized linear mixed models with R statistical software version 3.6.2 [25]. Since the data were zero–inflated and over–dispersed, as confirmed by the Shapiro test, a negative binomial distribution was employed [33]. “Dose”, “Trap distance” and “Household IDs” were treated as fixed effects, while sampling night was treated as a random effect. Separate analyses were performed for each mosquito species.

## Results

### Mosquitoes caught and identified

A total of 1568 mosquitoes were caught and identified, of which 783 (648 females, 135 males) were anophelines and 785 (577 females, 208 males) were culicines. Among the anophelines, 41.6% were primary malaria vector species, while 58.3% were secondary vector species (Table 1). The primary malaria vectors collected were *An. arabiensis* (92.9%; n = 560) and *An. funestus *sensu stricto (*s.s*.) (7.1%; n = 39). The three main secondary malaria vector species collected were *An. pharoensis* (72.7%; n = 618), *An. coustani* (12.9%; n = 113), and *Anopheles tenebrosus* (11.0%; n = 46) (Table 1)*.* As mirrored in the total collections, the physiological status of most female anophelines caught were unfed (551) followed by blood fed (54), and gravid mosquitoes (11) (Table 1). Among the culicines, 99.0% (n = 777) were *Culex* species while 1.0% (n = 8) were *Mansonia* and *Aedes* species (Table 1).

**Table 1:** Species, physiological state, and sex of the mosquitoes caught in traps baited with different doses of the synthetic cattle odour or a solvent control

Distance from dwelling											
		1.5 m					5.0 m				
		Dose									
Species	Sex & Physiological state	Control	0.003	0.01	0.03	0.1	Control	0.003	0.01	0.03	0.1
An. arabiensis*	Unfed	10	16	32	78	29	6	14	5	10	13
	Fed	2	0	8	11	3	1	0	0	4	1
	Semi-gravid	0	0	0	0	0	0	0	0	0	0
	Gravid	0	0	0	4	0	0	0	0	0	3
	Females (total)	12	16	40	92	32	7	14	5	14	16
	Males (total)	8	6	7	7	1	7	8	4	2	12
An. funestus s.s.*	Unfed	0	1	0	5	3	2	0	0	6	1
	Fed	0	0	0	0	0	1	0	0	0	0
	Semi-gravid	0	0	0	0	0	0	0	0	0	0
	Gravid	0	0	0	0	0	0	0	0	0	0
	Females (total)	0	1	0	5	3	3	0	0	6	1
	Males (total)	0	1	0	0	0	0	0	0	0	0
*An. leesoni*	Unfed	0	2	1	0	0	0	0	0	0	0
	Fed	0	0	0	0	0	0	0	0	0	0
	Semi-gravid	0	0	0	0	0	0	0	0	0	0
	Gravid	0	0	0	0	0	0	0	0	0	0
	Females (total)	0	2	1	0	0	0	0	0	0	0
	Males (total)	0	0	0	0	0	0	0	0	0	0
*An. rivulorum*	Unfed	1	0	0	0	0	0	0	0	0	0
	Fed	0	0	0	0	0	0	0	0	0	0
	Semi-gravid	0	0	0	0	0	0	0	0	0	0
	Gravid	0	0	0	0	0	0	0	0	0	0
	Females (total)	1	0	0	0	0	0	0	0	0	0
	Males (total)	0	0	0	0	0	0	0	0	0	0
*An. coustani*	Unfed	4	1	0	5	2	9	2	0	7	14
	Fed	0	0	0	0	0	0	0	0	0	5
	Semi-gravid	0	0	0	0	0	0	0	0	0	1
	Gravid	0	0	0	0	0	0	0	0	0	0
	Females (total)	4	1	0	5	2	9	2	0	7	19
	Males (total)	1	1	0	3	0	0	0	0	0	9
*An. pharoensis*	Unfed	14	31	29	12	10	12	17	77	38	26
	Fed	0	2	0	0	0	1	4	4	0	0
	Semi-gravid	0	0	0	0	0	0	0	2	0	0
	Gravid	0	0	0	0	0	0	0	2	0	2
	Females (total)	14	33	29	12	10	13	21	81	38	26
	Males (total)	4	2	8	4	4	10	10	12	4	0
*An. squamosus*	Unfed	0	0	0	2	0	0	2	0	0	1
	Fed	0	0	0	1	0	0	0	0	0	2
	Females (total)	0	0	0	3	0	0	2	0	0	3
	Males (total)	0	0	0	0	0	0	0	0	0	0
*An. ziemanni*	Unfed	0	0	0	0	0	0	0	1	0	0
	Fed	0	0	0	0	0	0	0	0	0	0
	Females (total)	0	0	0	0	0	0	0	1	0	0
	Males (total)	0	0	0	0	0	0	0	0	0	0
*An. tenebrosus*	Fed	0	0	0	0	1	0	0	0	2	1
	Females (total)	0	1	5	0	1	6	2	10	6	11
	Males (total)	0	0	0	0	0	0	0	0	0	0
*Culex spp.*	Females (total)	30	41	31	58	46	36	19	41	112	155
	Males (total)	11	12	16	24	6	13	5	34	25	62
*Mansonia spp.*	Females (total)	0	0	0	1	0	1	1	2	0	2
	Males (total)	0	0	0	0	0	0	0	0	0	0
*Aedes spp.*	Females (total)	0	0	0	0	0	1	0	0	0	0
	Males (total)	0	0	0	0	0	0	0	0	0	0

Control, heptane. An asterisk (*) indicates a primary vector

### Dose and distance from dwellings affect trap captures of malaria vectors

**Fig. 2:**
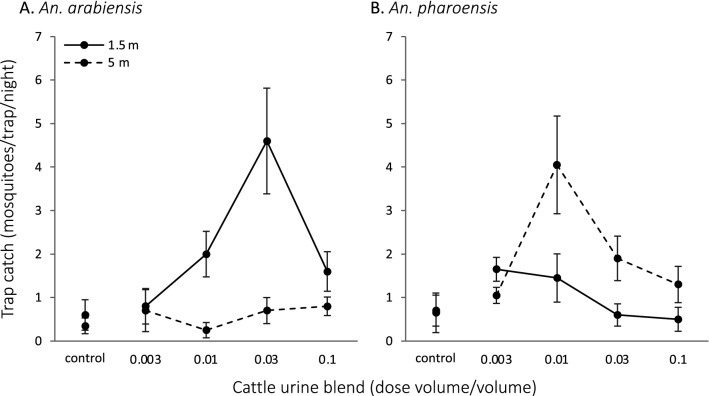
The mean nightly capture of **A** *Anopheles arabiensis* and **B** *Anopheles pharoensis* females in traps baited with different doses of the synthetic cattle urine odour or a solvent control, placed 1.5 and 5 m from human dwellings

The attractiveness of the synthetic cattle urine odour lure to both the primary and secondary malaria vector species varied with dose and trapping distance from the human dwelling (Table 1). For all of the primary malaria vectors caught, irrespective of physiological state, the lure attracted a significantly higher proportion of *An. arabiensis* (n = 229) relative to *An. funestus s.s.* (n = 16), irrespective of the dose and trapping distance (χ_1_^2^ = 86.512, P < 0.001). *Anopheles arabiensis* were caught more frequently next to the human dwellings (1.5 m) in a dose–dependent manner (GLMM: P = 0.001) compared to those further away (5 m; GLMM: P < 0.001) (Fig. 2A). While the number of *An. funestus s.s.* caught were too few to assess statistically, this species appeared to be caught more frequently at the higher doses tested, irrespective of distance from the human dwelling (Table 1). Of the secondary malaria vectors (Table 1), only *An. pharoensis* were caught in sufficient numbers for statistical analysis. This species was caught more frequently at a trapping distance of 5 m from the dwellings (GLMM: P = 0.044) in a dose-dependent manner (GLMM: P = 0.001) (Fig. 2B). *Anopheles pharoensis* appears to be more sensitive to the lure, as a higher number of individuals were caught at a lower dose than *An. arabiensis* (Fig. 2).

### Dose and distance from dwellings affect trap captures of Culex species

The attractiveness of the synthetic cattle urine odour lure to *Culex* species varied with dose, irrespective of trapping distance from the human dwellings (Table 1). *Culex* spp. were caught in a dose–dependent manner with more individuals caught in traps baited with the two highest doses (GLMM: P = 0.011 and P = 0.036, respectively).

## Discussion

The deployment of cattle urine–based attractants has the potential to improve outdoor mosquito surveillance and control tools [17–20]. In this study, the importance of trap placement in achieving the optimal efficacy of the cattle urine lure for targeting various species of both primary and secondary malaria vectors, as well as other vector and nuisance species, *e.g.*, *Culex* spp., is demonstrated. The findings, furthermore, demonstrate a species– and dose–dependent response towards the cattle urine lure. The results of this study are discussed in relation to further challenges to be addressed before this lure may be implemented in future integrated vector management programs.

The trapping distance from human dwellings significantly affected the number and species of mosquitoes caught in traps baited with the synthetic cattle urine lure. Females of the primary vector, *An. arabiensis*, both unfed and fed, were predominantly caught closer to the dwellings, while the majority of females of the secondary vector, *An. pharoensis*, unfed, fed and gravid, were caught at the farthest distance from the dwellings tested. This trend in the species profile caught close to the dwellings has been observed previously, which may have led researchers to conclude, erroneously, that cattle urine odour is not very attractive to *An. pharoensis* females [17–20]. In contrast to the anophelines, *Culex* spp. were caught in similar numbers irrespective of the distance from human dwellings. This finding emphasizes that the fine–scale spatial heterogeneity in the landscape is an important species-dependent driver of movement patterns of mosquitoes, as previously demonstrated for both anophelines and culicines [34, 35]. Thus, the efficacy of outdoor odour–baited traps relies on their proper deployment in the landscape, and a detailed understanding of the ecology of the mosquito species targeted.

*Anopheles arabiensis, An. pharoensis* and *Culex* spp*.* appeared to differ in their sensitivity to the synthetic cattle urine lure, with *An. pharoensis* likely being the most sensitive, and *Culex* spp. appeared to be attracted to the highest doses of the lure. Both *Anopheles* species were caught in lower numbers in traps that were baited with the highest dose of the lure, demonstrating a similar behavioural response as that of tsetse flies to various doses of cattle urine odour [36]. The rationale for the observed species–specific variation in behavioural response is likely due to differences in perception of the bioactive volatile organic compounds in the synthetic cattle urine odour. Whether adaptive selection has affected the association between select mosquito species and cattle urine remains to be explored.

## Conclusions

This study provides additional essential information on the synthetic cattle urine lure, developed to target exophilic and exophagic malaria vectors outdoors, for which efficient surveillance and control tools are currently lacking. The efficacy of the lure was dependent on both dose and the placement of the traps used to capture select mosquito species, as demonstrated here. While additional testing of the lure in other geographic regions, with other landscape and vector species profiles, may be required, the main obstacles to be overcome include the high regulatory burden imposed on semiochemical tools used for integrated vector control. Moreover, this novel integrated vector management technology must be reviewed, evaluated, and recommended by the World Health Organization prior to being adopted by national programmes.

## Data Availability

All data generated and/or analysed during this study are included in this published article. The materials collected during the study are available from the corresponding author on reasonable request.

## Abbreviation

GLMM: Generalized linear mixed effect model
